# *Xylella fastidiosa* gene expression analysis by DNA microarrays

**DOI:** 10.1590/S1415-47572009005000038

**Published:** 2009-05-01

**Authors:** Regiane F. Travensolo, Lucia M. Carareto-Alves, Maria V. C. G. Costa, Tiago J. S. Lopes, Emanuel Carrilho, Eliana G. M. Lemos

**Affiliations:** 1 Departamento de Tecnologia, Faculdade de Ciências Agrárias e Veterinárias de Jaboticabal, Universidade Estadual Paulista Júlio de Mesquita Filho, Jaboticabal, SPBrazil; 2 Instituto de Química de São Carlos, Universidade de São Paulo, São Carlos, SPBrazil

**Keywords:** * Xylella fastidiosa*, DNA microarray, gene expression

## Abstract

*Xylella fastidiosa* genome sequencing has generated valuable data by identifying genes acting either on metabolic pathways or in associated pathogenicity and virulence. Based on available information on these genes, new strategies for studying their expression patterns, such as microarray technology, were employed. A total of 2,600 primer pairs were synthesized and then used to generate fragments using the PCR technique. The arrays were hybridized against cDNAs labeled during reverse transcription reactions and which were obtained from bacteria grown under two different conditions (liquid XDM_2_ and liquid BCYE). All data were statistically analyzed to verify which genes were differentially expressed. In addition to exploring conditions for *X. fastidiosa* genome-wide transcriptome analysis, the present work observed the differential expression of several classes of genes (energy, protein, amino acid and nucleotide metabolism, transport, degradation of substances, toxins and hypothetical proteins, among others). The understanding of expressed genes in these two different media will be useful in comprehending the metabolic characteristics of *X. fastidiosa,* and in evaluating how important certain genes are for the functioning and survival of these bacteria in plants.

## Introduction

*X. fastidiosa* (Wells *et al.*, 1987) belongs to the gram-negative group and is restricted to the xylem vessels of host plants. It has been associated with diseases that affect diverse plant species, some of which economically important, these including alfalfa, almonds, blackberries, coffee, citrus fruits, grapes, peaches, pears, plums and certain ornamental plants (Hopkins, 1989). Citrus Variegated Chlorosis (CVC) was first detected in Brazil in 1987, and currently constitutes a serious threat to the Brazilian orange juice industry, since it is present in the main cultivation areas, being responsible for significant losses in orange production (Rossetti *et al.*, 1990). In 2000, a consortium of laboratories in São Paulo State published the *X. fastidiosa* isolate 9a5c sequenced genome. A main chromosome (2,679,305 base pairs) and two other plasmids (51,158 and 1,285 base pairs) were sequenced, these presenting a total of 2,905 genes, from which half presented similarity with unknown protein functions (Simpson *et al.*, 2000).

Understanding the complete genome sequence was a substantial advance towards comprehension of metabolic and replicate characteristics, and for starting the first approach in determining pathogenicity mechanisms. Papers published recently have explored the information generated by genomic sequencing, highlighting a series of hypotheses related to the functioning of energy metabolism, nutrient transport, adherence, aggregation, toxicity, the secretion of pathogenicity factors, intercellular interactions, iron homeostasis, antioxidant responses and other important pathogenicity mechanisms (Simpson *et al.*, 2000; Keen *et al.*, 2000; Dow and Daniels, 2000; Lambais *et al.*, 2000; Silva *et al.*, 2001; Leite *et al.*, 2002; Meidanis *et al.*, 2002).

It is known that *X. fastidiosa* demands a complex medium for its *in vitro* development (Holt *et al.*, 1994). With *X. fastidiosa* genome sequencing, the possible genes involved in bacterial metabolism have become known, and as a result, a defined and adequate medium for cultivating *X. fastidiosa*, known as XDM_2_ (*Xylella* defined medium), was set up (Lemos *et al.*, 2003). The components of this medium have been included based on metabolic pathways found with the help of information obtained from the *X. fastidiosa* genome. XDM_2_ contains glucose, vitamins (biotin, thiamine, pyridoxine hydrochloride and nicotinic acid) and amino acids (serine, methionine, asparagine and glutamine), as well as iron, phosphate, sulfate and myo-inositol. The XDM_2_ medium has made it possible to cultivate *X. fastidiosa* more successfully than by using the complex BCYE modified media (Campanharo *et al.*, 2003), which presents in its formulation only yeast extract and an ACES buffer. Furthermore, differences among *X. fastidiosa* isolates, obtained from various host plants, have been observed. These are related to their cultivation in media of different compositions, thereby indicating the existence of genetic variability within this group of bacteria (Hopkins, 1989).

The present accumulation of information with the sequencing of genomes from various organisms has offered an enormous opportunity to understand the biological functions of many genes, previously described as unknown (Lashkari *et al.*, 1997). Furthermore, microarray technology (Shalon *et al.*, 1996) provides a simultaneous way for immediately monitoring the expression of several genes. In practice, it is possible to arrange about 6,000 elements (genes) in an area of less than 1.8 cm^2^. A nearly complete collection of 4,290 *Escherichia coli* open reading frames (ORFs) was obtained for analyzing the expression ratio of this bacterium when cultivated in two different media (minimal and rich). Bacteria cultivated in a rich medium presented accelerated multiplication, the higher number of genes significantly expressed being related to the translation apparatus. On the other hand, bacteria cultivated in the minimal medium showed elevated expression of many of those genes involved in biosynthetic pathways, mainly in the amino acids (Tao *et al.*, 1999).

The aim of this work was to develop a DNA microarray analysis set, and undertake a transcriptional study of those genes related to the metabolism of the *X. fastidiosa* 9a5c strain isolated from citrus fruits, when cultivated under two distinct conditions, BCYE (complex media) and XDM_2_ (defined media).

**Figure 1:**
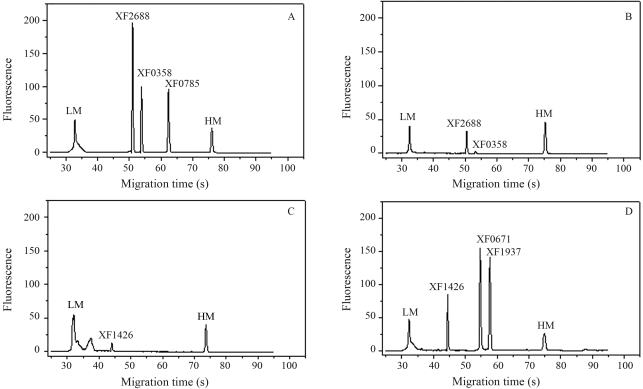
Microchip electrophoresis of the reverse transcription-polymerase chain reaction (RT-PCR) products of six ORFs (XF2688, XF0358, XF0785, XF1426, XF0671 and XF1937). A and C: genes expressed in XDM_2_ media; B and D: genes expressed in BCYE media. LM: lower marker. HM: higher marker.

## Materials and Methods

### Cultivation conditions

For genomic DNA extraction, *X. fastidiosa* isolate 9a5c was cultivated in ‘Petri dishes' containing BCYE medium (Wells *et al.*, 1981) at 28 °C for six days. For RNA extraction, the bacteria were cultivated for four days at 30 °C in a 250 mL Erlenmeyer flask containing 30 mL of either liquid XDM_2_ medium (Lemos *et al.*, 2003) or liquid modified BCYE (Campanharo *et al.*, 2003) under shaking conditions (140 rpm).

### Isolation of genomic DNA and total RNA

Genomic DNA was extracted according to the described methodology (Ausubel *et al.*, 1987) with a modification that includes a step with RNAse treatment as follows: 200 μg/mL, 1.5 h at 37 °C. The methodology used for RNA extraction (Chomczynski and Sacchi, 1987) involved a monophasic solution of phenol and guanidine isothiocyanate (Trizol - Invitrogen). The RNA samples treated with DNAse I were purified through the NucleoSpin^®^ RNA II BD Bioscience kit (Clontech), resuspended in H_2_O_DEPC_ and stored at -80 °C.

### Synthesis of fluorescent cDNA from total RNA

The construction of fluorescent cDNA for hybridization reactions was by means of a CyScribe cDNA Post Labeling kit (Amersham Biosciences) with 30 μg of RNA and 15 μg of random primers (Amersham Bioscience). The reverse transcriptase reaction occurred at 37 °C for 3 h in a programmable thermocycler (PTC-100 Programmable Thermal Controller - MJ Research, Inc.). As the control of the transcriptase reaction, 1 μL of the synthetic RNA from the Lucidea Universal ScoreCard kit (Amersham Biosciences) was used. The reaction was neutralized with 20 μL of 2 M of HEPES, and the cDNAs were purified through precipitation with 3 M of sodium acetate and 75 μL of 100% v/v ethanol, and kept at - 20 °C overnight. After centrifuging and washing with 70% ethanol, the cDNA was resuspended in 30 μL of CyDye diluted in 0.1 M of bicarbonate of soda, pH 9.0. The sample was kept in the dark at 25 °C for 1 h, and the labeling reactions were stopped by the addition of 15 μL of 4 M of hydroxylamine for 15 min at 25 °C. The sample was then resuspended in 400 μL of a TE buffer (10 mM of Tris-HCl pH 8.0, 1 mM of EDTA) and concentrated in a Microcon-type column -YM30 (Millipore). The efficiency of the reading was monitored by measuring absorption at 260 nm (for DNA concentration), 550 nm (for Cy3) and 650 nm (for Cy5).

### Amplification of *X. fastidiosa* genes

Primers were used to amplify the 2,600 ORFs of the *X. fastidiosa* genome. These primers were built for both forward and reverse directioning, ranging from 16 to 19 nucleotides in length and from 48 °C to 57 °C in Tm (melting temperature). PCR reactions were done in a total volume of 100 μL containing a PCR buffer (50 mM of KCl, 200 mM of Tris-HCl pH 8.4), 2 mM of MgCl_2_, 10 mM of dNTP, 2 U of Taq DNA polymerase (all from Invitrogen), 5 pmol of each primer, 60 ng of genomic DNA and pure sterile water to complete the volume. The reactions were performed in a programmable thermocycler (PTC-100 Programmable Thermal Controller-MJ Research, Inc.), the following programs being used: 94 °C for 2 min, 35 cycles (94 °C for 1 min, 58 °C for 1 min and 72 °C for 1 min and 30 s) and a final step at 72 °C for 5 min. All the products were analyzed by electrophoresis in agarose gels of 1.5% w/v in TEB containing 0.5 μg/mL ethidium bromide. Amplifications were considered successful when only one product was visualized and when the size of the expected product varied from 300 to 1000 bp.

### Microarray construction

The amplified products were suspended in 50% v/v DMSO in a final concentration of 100 to 300 ng/μL and arranged in duplicate at a distance of 250 μm in glass slides treated with aminosilane (Corning). Printing of microarrays was done by a robot model GMS 417 Arrayer (Affymetrix Inc.). After printing, the DNAs were re-hydrated (42 °C for 10 s), dried (70 °C for 1 min) and fixed in a UV camera cross-link (1300 x 100 μJ cm^2^). The slides were kept at 70 °C for 2 h and then stored under vacuum at room temperature. Genetically distant negative controls were also included in this array, these consisting of human genes (pHUM1 and pHUM7) and plant genes (707050B11 - Rubisco), as well as synthetic controls from various species (human, mouse, *Arabidopsis* spp., Archaeabacteria and *E. coli*) obtained by the Lucidea Universal ScoreCard kit (Amersham Biosciences).

### Hybridization and washing

Hybridization and washing were carried out in a GeneTac Hybridization (Genetic MicroSystems) device. Initially, slides containing the microarrays were denatured at 65 °C for 5 min. A solution containing 8 μL of blocking liquid (Amersham Biosciences RPN 3601), 19 μL of SSC 20x, 5.5 μL SDS 2% w/v and 100 pmol of cDNA marked with the fluorescent dyes Cy3 and Cy5, totaling 110 μL, was denatured at 95 °C for 2 min, deposited on the slide and kept for 12 h at 42 °C. After hybridization, the slides were washed at 25 °C in the following solutions: 2x SSC/SDS 0.5% w/w, 0.5x SSC and 0.05x SSC. All washing-steps consisted of 10 cycles with 10 s of solution flux and 20 s of incubation. The slides were then dried for 15 min and submitted to fluorescence detection.

### Image acquisition and data analyses

The slides were submitted to fluorescence reading in a model GMS 418 Arrayer Scanner (Affymetrix Inc.) under different wavelengths - 550 nm (Cy3) and 650 nm (Cy5). The location and identity of each gene on the slide was defined in a text file, created with the aid of the CloneTracker2 program (BioDiscovery). The signal was quantified through ImaGene software (v. 4.1, BioDiscovery), in which two images from the Cy3 and Cy5 fluorescent dyes were overlapped and the spots classified according to morphology and intensity. The computer displays an electronic symbol as a false-color image where a red or green spot corresponds to expression of a gene in sample 1 or 2, respectively, while a yellow-orange spot indicates that the gene was expressed at similar levels in both samples. For transformation of data, the background signal was discounted from the signal of each spot using the local background obtained by the GeneSight (BioDiscovery) program. The transformation sequence included background correction, omitted flagged spots, combined replicates and floor, by adding a shifted Log transformation and ratio.

Data obtained from the intensity ratio of the signal measured by the Cy5 (experiment) over the Cy3 (control) were normalized according to the average intensity of the total signal. We used all the genes in our dataset to calculate normalization since this assumes that the majority of the measured genes were not differentially regulated. The normalization procedure is a suitable approach for minimizing variations so that a common base for comparison is established. There are a number of reasons that justify data normalization, these including unequal quantities of starting RNA, differences in labeling or detection efficiency among the different fluorescent dyes used, and systematic bias in measured expression levels (Quackenbush, 2002). However, current normalization methods are not applicable to all conditions. Normalization can be carried out in several different ways, such as within the slide in order to adjust dye incorporation efficiency, between two slides for dye swap experiments and across slides for repetition of the same experiments (Yang *et al.*, 2001). In the latter case, application would be to the entire data set (overall normalization), instead of to a particular physical data subset or sub grid (local normalization).

Final intensity of hybridization was determined in all the experiments from six replicates per data point, and is representative for three independent determinations (slides) from each media culture. Replicates in duplicate within each slide were combined by the median of their values, whereupon statistical analysis was carried out using the SAM method (Significance Analysis of Microarrays). This method is based on *t*-test statistics and is employed to calculate the false discovery rate (FDR) and gene error chance (*q*-value) (Tusher *et al.*, 2001). Significant variations in expression of those genes related to *X. fastidiosa* metabolism were compared when cultivated in liquid modified BCYE and liquid XDM_2_ median.

### Detection of cDNA by micro-chip electrophoresis

The reverse-transcription step for generation of cDNA was performed in a final volume of 20 μL using 1 μg of total RNA digested with 0.5 μM DNAse I. The random primer (1 μM) and digested RNA were denatured for 5 min at 70 °C, then immediately cooled on ice for another 5 min and added to a 15 μL RT mix containing 2.0 mM of dNTPs, 3 mM of MgCl_2_, 1x RT buffer and 1 μL of ImProm-II RT (all from Promega, Madison, WI, USA). The mixture was incubated for 5 min at 25 °C, 60 min at 40 °C, and 15 min at 70 °C. PCR reactions were set up in 10 μL total reaction volume containing 1x PCR buffer, 2 mM of MgCl_2_, 10 mM of dNTP, 2 U of Taq DNA polymerase, 5 pmol/L of each primer (Table 1) and 1.5 μL of cDNA. The reactions were performed in a programmable thermocycler (PTC-100 Programmable Thermal Controller-MJ Research, Inc.) under the following conditions: 94 °C for 2 min, 35 cycles (94 °C for 1 min, 58 °C for 1 min and 72 °C for 1 min and 30 s) and a final step at 72 °C for 5 min. All products were analyzed by using the Bioanalyzer 2100 (Agilent Technologies, Waldbronn, Germany) in conjunction with the LabChip DNA 500 kit, according to manufacturer's instructions.

## Results and Discussions

### Synthesis of fluorescent labeled cDNA

In order to verify gene expression differently, we analyzed the growth of *X. fastidiosa* from four days after culture in two different liquid media, modified BCYE and XDM_2_ (Table 2). Based on the genetic analysis of the *X. fastidiosa* genome, Lemos *et al.* (2003) developed certain media with a defined composition, whereby the growth abilities of these bacteria were evaluated in both liquid media and on solid plates. *X. fastidiosa* growth was compared in XDM_2_ defined media as well as in BCYE by measuring cell turbidity and protein content for 14 days at 28 °C under shaking conditions. The authors observed that, after 14 days, the growth rate of bacteria on complex media, such as BCYE was substantially lower than in XDM_2_, and that, in the latter, the strains grew equally well both in liquid and on solidified media. However, after four days (96 h) cell turbidity and protein content were similar for both.

Preparation of fluorescent labeled cDNAs was carried out by total RNA extraction from *X. fastidiosa* growth after four days in liquid modified BCYE and liquid XDM_2_ media, its concentration being determined by absorbance measurement at 260 nm (A_260_). RNA integrity was checked by formaldehyde agarose gel electrophoresis, where the occurrence of two ribosomal subunit bands (23S and 16S containing of 2.9 and 1.5 kb, respectively) was examined (data not shown).

Fluorescent labeled cDNAs were prepared from total *X. fastidiosa* RNA by reverse transcription. Total RNA was used since most of the mRNAs produced by bacteria do not have a poly (A) tail and are difficult to separate. Labeling efficiency by reverse transcription depends on incorporation efficiency and on the amount of specific nucleotides present in a particular mRNA species. The labeling kit used was developed as a two-step procedure. The first step involves the incorporation of amino allyl-dUTP (AA-dUTP) during cDNA synthesis by using an optimized nucleotide mixture. The second step involves chemically labeled amino allyl-modified cDNA using CyDye NHS-esters. Coupling reactions of amino allyl-modified cDNA were performed separately with Cy3 and Cy5 and both targets were combined in the hybridization solution. The amount of target used for hybridization depends on array format and labeling method. Targets containing 100 pmol of incorporated fluorescent dye were employed. Such an amount was calculated from the formulas, (OD_550_ x dilution factor x total volume)/0.15 for Cy3 and (OD_650_ x dilution factor x total volume)/0.25 for Cy5, where the obtained values are in pmol.

### Arraying amplified *X. fastidiosa* genes

DNA arrays were developed through the synthesis of 2,600 amplicons using pairs of primers related to each of the *X. fastidiosa* genome ORFs. Amplified ORFs were set in a concentration varying from 100 to 300 ng/μL, with fragment-size also varying from 300 to 1,000 bp. The DNA arrays were composed of amplicons that did not need to be purified. The spots were printed in duplicate with a 250 μm distance between each, to a total of 5,200 spots, including positive and negative controls. Studies in microarray gene expression analysis, using unpurified amplified products, emphasized non-significant differences between purified and unpurified PCR products, showing a low alteration level in the hybridization signal (6%) in the latter, when compared to the purified version (Diehl *et al.*, 2002).

The comparison of the expression of *X. fastidiosa* 9a5c genes when isolated from bacteria cultivated in the two different media (liquid modified BCYE and liquid XDM_2_) was carried out by using SAM software which develops a statistical evaluation of probes' hybridization patterns. The significance of gene expression differences was calculated by the ratio of median fluorescence intensities for each condition, having as parameters a ratio difference of at least 1.5x together with a threshold Δ of 0.49514. Missing data points were estimated with a *K*-Nearest-Neighbor imputator equal to 10. The test was undertaken with response format type paired data with a false discovery rate (FDR) of less than 0.5%.

Data analysis resulted in a 0.42 false positive rate (FSN - False Significant Number) and 0.31 of false discovery genes, thus demonstrating that 99.69% of our experiments present positive results and only 0.31% are false. Among the analyzed genes, approximately 5.15% (134) were detected as differentially expressed in both studied conditions. 30 of these (22.4%) showed higher expression in the BCYE medium and 104 (77.6%) in the XDM_2_ (Table 3). All gene-chip data can be found in the Gene Expression Omnibus (GEO) Repository under accession number GPL7554.

According to the results obtained through microarray analysis, bacteria cultivated in XDM_2_ medium expressed a higher number of significant genes than those cultivated in BCYE modified medium. This was expected, since the XDM_2_ defined medium offers a smaller variety of nutrients than the BCYE complex medium. These differences in gene expression patterns were analyzed in detail, as described below.

### Genes involved in energy metabolism

Significantly high expression levels were observed when bacterial cells were cultivated in the XDM_2_ medium for the following genes: *gapA* (glyceraldehyde-3-phosphate dehydrogenase), *rfbC* (DTDP-4-keto-L-rhamnose reductase), *mdh* (malate dehydrogenase), *odhA* (oxoglutarate dehydrogenase), *pfkA* (6-phosphofrutokinase), *gcvT* (glycine cleavage T protein), *fumB* (fumarate hydratase), *az1* (electron transfer protein azurin I), *tpiA* (triosephosphate isomerase), *yahK* (alcohol dehydrogenase), *petB* (ubiquinol cytochrome C oxidoreductase), *atpG* (ATP synthase) and *acnB* (aconitate hydratase 2), whereas, a higher expression was observed for the following genes in those cells cultivated in the BCYE medium: *pdhB* (dihydrolipoamide acetyltransferase) and *pykA* (pyruvate kinase type II).

The functionality of the glycolytic pathway in *X. fastidiosa* was evaluated (Facincani *et al.*, 2003) by studying the enzymes phosphoglucose isomerase, aldolase, glyceraldehyde-3-phosphate dehydrogenase and pyruvate kinase from the glycolytic pathway, and glucose-6-phosphate dehydrogenase from the Entner-Doudoroff, followed by cloning and expression studies of the enolase gene and determination of its activity. These studies showed that *X. fastidiosa* does not use the glycolytic pathway to metabolize carbohydrates. As a result, no enzymatic activity was detected for enolase, aldolase and glyceraldehyde-3-phosphate dehydrogenase, this suggesting that *X. fastidiosa* may be using the Entner-Doudoroff pathway to produce pyruvate as an alternative. Nevertheless, an increase in gene expression of those enzymes related to the glycolytic pathway in the cultivated cells was detected through microarray analysis, this regardless of the supporting XDM_2_ medium. This set of genes codes for 6-phosphofrutokinase, glyceraldehyde-3-phosphate dehydrogenase and triosephosphate isomerase (data collected from the cells raised on XDM_2_), as well as for pyruvate kinase (data collected from cells raised in a BCYE modified medium).

In this work we observed the expression of dihydrolipoamide acetyltransferase, malate dehydrogenase, oxoglutarate dehydrogenase, fumarate hydratase, aconitate hydratase 2, electron transfer protein azurin I and ATP synthase, which act in the reduction of pyruvate, the citric acid cycle, electron transportation, and the production of ATP. The presence of certain TCA cycle enzymes and of the respiratory chain contributes to the hypothesis that *X. fastidiosa* uses cell respiration to obtain energy from glucose.

### Transport related genes

A total of 140 genes that code for proteins related to the transport of a number of biological molecules were identified in the *X. fastidiosa* genome, these representing 4.8% of all ORFs (Simpson *et al.*, 2000). Among these genes, 19 were considered differentially expressed in this comparison, 14 being detected as higher expressed in XDM_2_ medium conditions and five in BCYE. These genes refer to the transport of anions, cations, carbohydrates, peptides, proteins and substances related to secretory pathways. Genes involved with the secretion of peptides and proteins (*xpsH* and *secY* in XDM_2_ medium, *xpsD* and *secA* in BCYE) were expressed in both media, these four being related to general secretion and Sec systems. The *xpsH* and *xpsD* genes expressed code for external membrane proteins that act in the General Secretory Pathway (GSP) Type II. The secreted enzymes in this pathway include polygalacturonate lyase, endoglucans, α- amylase and proteases (Gough *et al.*, 1988; Hu *et al.*, 1992). On the other hand, in gram-negative bacteria such as *X. fastidiosa*, macromolecules, which include excreted enzymes, toxins and structures from the surface of the cell, need to pass through both the internal and external membranes before reaching the surface of the cell (Hu *et al.*, 1998; Fekkes and Driessen, 1999). The Sec system involves an integral membrane heterotrimer, SecYEG, also known as the translocation complex, which acts together with a homodimeric protein, SecA, which is ATP-dependent. A characteristic of this mechanism is that proteins are translocated in extended conformation, and are frequently bound to SecB or another cytoplasmic chaperonin for proper folding (Berkes *et al.*, 2000).

Most bacteria have other secretory pathways that are distinct from the Sec apparatus (Weiner *et al.*, 1998). One of these Sec-independent pathways was named the TAT system (Twin Arginine translocation system) (Sargent *et al.*, 1998), due to precursors activating the pathway through a signal peptide that includes two consecutive arginine residues. The characteristic of the TAT pathway is that it works to transport folded proteins of various sizes through the cytoplasmatic membrane (Berkes *et al.*, 2000). The *tatD* gene, which is cotranscribed with *tatA*, *tatB* and *tatC*, was expressed under BCYE medium conditions, but apparently does not have any effect on translocation of those proteins containing arginine residues, since it codes for a cytoplasmatic protein with DNAse activity with no discernible role in tat translocation (Wexler *et al.*, 2000).

Other more expressed genes in the XDM_2_ medium were *malG*, *ynhE*, *yecS*, *algS*, *yheS* and *ccmA*, which code for proteins belonging to the ABC transport system. This secretory system depends on the mediation of ABC proteins, consisting of three cell wall proteins, two internal membrane proteins and an external membrane polypeptide (Binet *et al.*, 1997). The *malG* and *algS* genes are related to the ABC sugar-transportation system. These two genes were significantly expressed in cells cultivated in the XDM_2_ medium, in which glucose was found at a concentration of 10 g/L, as compared to cells cultivated in BCYE medium, with no glucose at all (Lemos *et al.*, 2003). Glucose is the only carbon source found in XDM_2_ which is transported and used for energy production within *X. fastidiosa* cells, and whose intermediary compounds are involved in glycolysis, the citric acid cycle and the electron-transportation chain, since this microorganism has all the genes related to such energy-associated cycles.

### Genes involved in membrane components and surface structures

Six genes that code for proteins related to fimbriae were expressed in both analyzed cultivation media: (XF2542, *mrkD*, *pilU*, *pilP* and *pilY-1* in the XDM_2_ medium and *pilQ* in the modified BCYE medium). The *pilP*, *pilY-1* and *pilQ* genes are related to type IV fimbriae involved in fimbriae biogenesis, whereas *pilU* and XF2542 are supposedly responsible for fimbriae retraction and extension, a mechanism known as twitching motility. XF2542 is similar to the subunits of *Xanthomonas* spp. and *Pseudomonas* spp. type IV fimbriae. A specific gene expression regulation mechanism of type IV fimbriae was observed in different cultivation conditions, suggesting that this is an important factor for *X. fastidiosa* survival (Smolka *et al.*, 2003).

In the XDM_2_ medium, transcripts of *mrkD* (*fimA* family) genes involved in the adherence of *X. fastidiosa* bacteria were detected. This protein is similar to others found in a number of bacterial species that infect plants, animals and human beings (Ojanen-Reuhs *et al.*, 1997). It is considered to be a key mediator for adhesion and mobility, being an important virulence factor. However, isolated specimens with mutations in *fimA*^-^ and *fimF*^-^ became pathogenic when inoculated into vine plants (Feil *et al.*, 2003). Hitherto, these two genes have been discarded from involvement in the mechanisms of pathogenicity.

As far as membrane components are concerned, four genes were found expressed in the XDM_2_ medium (*dc-14*, XF0881, *mreB* and *murD*) and only one in the BCYE modified medium (*mopB*). These genes were related to proteins of the internal and external membrane, besides cell wall biogenesis. The *mreB* and *murD* genes code for proteins linked to the production of peptidoglycan, which is the main component of bacterial cell walls, and consists of the heteropolymers of N-acetylglucosamine and N-acetylmuramic acid.

The *X. fastidiosamopB* gene is very similar to the OprF porin protein that belongs to the OmpA family from *Pseudomonas* spp., and which is involved in xylem endophytic bacteria growth and survival ability in low osmolarity niches (Rawling *et al.*, 1998). The *mopB* gene can be pinpointed as an interesting target, since *X. fastidiosa* survives in a low osmolarity environment when inside xylem vessels.

### Genes involved in RNA, DNA and nucleotide metabolism

The analyses revealed higher gene expression in bacteria cultivated under XDM_2_ conditions for those genes related to RNA and DNA metabolism (*vacC*, *metG*, *holA*, *holB*, *recG* and *mutY*). On the other hand, for bacteria cultivated in a modified BCYE medium, only *gltX*, *ilaIIA* and *tyrS* genes were considered as showing significant and high expression levels (Table 3). Furthermore, *X. fastidiosa* showed a higher rate of cell multiplication, when grown in XDM_2_ medium than in, modified BCYE medium (Lemos *et al.*, 2003). This is in accordance with the levels of expression of those genes related to nucleic acid metabolism, since a larger number of these genes were expressed under XDM_2_ conditions. Thus a larger number of ribosomes and a higher speed of protein synthesis were observed for accelerated cell division cycles (Grunberg-Manago, 1996), as happens in the XDM_2_ medium.

The significant expression difference for *X. fastidiosa* genes, when cultivated in the XDM media series, was essentially related to the production of ribosomal proteins (Nunes *et al.*, 2003). This defined medium presents only glycerol and glutamic acid in its composition, or rather the XDM_2_ precursors used in this paper. The authors suggest that the majority of *X. fastidiosa* genes may be under the control of constitutive promoters, which are induced under nutrient limiting conditions, this representing an important step towards the adaptation of such a bacterium to the adverse conditions found within the xylem vessels of infected plants.

Three ORFs related to nucleotide biosynthesis were expressed in the XDM_2_ and modified BCYE media: phosphoribosylaminoimidazole carboxylase (*purE*), gluconolactonase precursor (SCF 11.04) and 5-phosphoribosyl-5-aminoimidazole synthetase (*purM*). The *purE* and *purM* genes are responsible for synthesis of purine ribonucleotides, while SCF11.04 acts on the biosynthesis of nucleosides. This reaction is part of purine biosynthesis, starting with the metabolic precursors, ribose-5-phosphate, CO_2_ and NH_3_. All the pathways for synthesis of purinic and pyrimidinic nucleotides have already been described for *X. fastidiosa* (Simpson *et al.*, 2000).

### Genes involved in the biosynthesis of amino acids and proteins

Through the analysis of those genes related to amino acid biosynthesis, it was possible to observe that *X. fastidiosa* is able to synthesize certain amino acids such as aspartate, cysteine, glutamate, histidine and metionine. Most microorganisms can uptake amino acids from their cultivation medium and oxidize them to sustain energy levels, as required by metabolic conditions (Nelson and Cox, 2002). *X. fastidiosa* presents high biosynthetic capacity, this probably resulting from its success in colonizing the xylem vessels of a number of host plants (Simpson *et al.*, 2000). However, xylem fluid contains a low concentration of organic composts (available energy sources), although it presents a high concentration of amino acids such as glutamine and asparagine (Raven, 1984). Glutamine and arginine are important in the composition of the XDM_2_ medium, as sources of nitrogen and in helping *X. fastidiosa* cells to reach the end of their exponential growth phase in less generation-time (Lemos *et al.*, 2003).

Genes related to amino acid biosynthesis were found in both culture media at various expressed levels. Two implications arise from this analysis. The first confirms that the TCA cycle is active, since it generates the intermediaries for amino acid biosynthesis from the glucose oxidative degradation pathway. The second implication is that the source of amino acids in both media, mainly in XDM_2_ (which contains arginine, glutamine, metionine and serine), can be used in protein synthesis as well as for supplying the carbon skeleton: a) to replace intermediaries of TCA cycle components in anaplerotic reactions and b) for synthesis of the other amino acids.

The operon *sspA*-*sspB* expression in the XDM_2_ medium was similar to that observed in *E. coli* during the stationary phase of the growth curve and under carbon, amino acids and phosphate limiting conditions (Willians *et al.*, 1994). This operon expression level during the four days of *X. fastidiosa* cultivation shows that the active metabolism of the bacterial cells in the XDM_2_ medium results in the consumption of nutrients up to cells entering the stationary growth phase.

Analyses revealed higher gene expression for the *pspB* gene which codes serine protease, in bacteria cultivated in the XDM_2_ medium (Table 3). Serine protease is not secreted via a type I pathway, but belongs to the autotransporter family of secreted proteins (Chabeaud *et al.*, 2001). Many proteins belonging to the autotransporter family are involved in adhesion or auto-aggregation, even though several of them possess the active-site motif of serine protease (Henderson *et al.*, 1998).

### Genes involved in cofactors, prosthetic groups and regulatory functions

Through genomic analysis, it was suggested that vitamins like thiamin, biotin, nicotinic acid and pyridoxine are synthesized by *X. fastidiosa* cells (Simpson *et al.*, 2000). The significant expression of genes related to the prosthetics groups, cofactors and vitamins, such as those involved with riboflavin (*ribA* and *ribD*), biotin (*bioA* and *bioB*), pantothenate (*panC*), porphyrin (*hemB* and *hemF*), folic acid (*folK*) and thiamin (*thiL*), may suggest that the concentrations used in media composition were insufficient to sustain growth, and that the decrease in these levels was responsible for their synthesis. On the other hand, the XDM_2_ medium containing only biotin as a vitamin source and in a 0.2 mg/L concentration was efficient enough to maintain bacterial cell growth (data not shown). The regulatory functions category presented six highly expressed genes in the XDM_2_ medium (*sspA*, *sspB*, *tctD*, *algR*, *colR*, *pilR*) in comparison to only one gene (*phoR*) in the BCYE modified medium. Since the functions of most of these systems are still unknown, it is believed that the organisms in which they are expressed may show a higher level of adaptive answers to certain environmental changes, situations in which the two component systems are induced (Stock *et al.*, 1989).

The *phoR* gene is induced by phosphate limitation (Hullet, 1996), being significantly expressed only in the BCYE modified medium which does not include ferric pyrophosphate, a possible source of phosphate, in its composition. The significant expression of *pho* genes clearly indicates the need to include other sources, for this bacterium to make use of phosphorus for growth.

### Genes involved in molecule degradation

Four expressed genes were related to the category of degradation, with emphasis on *est* (esterase), *lipP* (lipases) and *dhaA* (haloalkane dehalogenase). A correlationp between lipase concentration and the production of biofilms might be linked to adhesion and construction of the latter (Smolka *et al.*, 2003). Lipases hydrolyze ester bonds between the insoluble triacilglycerides interface and the aqueous phase where the enzyme is dissolved (Anthonsen *et al.*, 1995). In *Candida albicans*, LIP family lipases are expressed and eliminated during the infection cycle, and it is believed that they contribute to the survival and virulence of this organism in human tissues (Hube *et al.*, 2000).

### Genes related to hypothetical and conserved proteins

Approximately 30% (40 genes) of the 134 differentially expressed genes did not present homology with sequences deposited in GenBank. The involvement of hypothetical and conserved proteins, for which functions in other organisms have not yet been described, should be taken as an indication of significant differences in the metabolism of this phytopathogen.

### Categories of genes expressed only under XDM_2_ cultivation conditions

The expression of genes related to the toxin categories (*frpC*, *gaa* and *cvaB*), functions related to plasmids (*taxC*), phago (*int*), cell division (*ftsW*) and others (*bcp*), were only observed in XDM_2_ medium growth conditions. Among these categories, toxins that can be depicted belong to the RTX family (gene *frpC*) and the production of colicin V (gene *cvaB*). The *cvaB* gene from *E. coli*, together with two other genes, *cvaA* and *tolC*, mediates the elimination of colicin V (ColV) to the outer part, so as to act within target cells, thereby destroying them by membrane rupture (Zhong and Tai, 1998).

### Categories of genes expressed only in BCYE modified cultivation conditions

The expression of the *fadG* gene can be assigned to biosynthesis of fatty acids and was observed to be high under the BCYE modified medium conditions. The *E. colifab* genes presented higher expression levels in a rich medium, thereby suggesting that the regulation of phospholipid biosynthesis genes might be dependent on the speed of growth, since these genes need a higher number of membrane compounds (Tao *et al.*, 1999). However, studies with *X. fastidiosa* demonstrated that growth was higher in the XDM_2_ medium than in the BCYE during the 14-day period (Lemos *et al.*, 2003). Thus, it is possible that *fab* gene expression is mediated by one or more signal molecules found in the modified BCYE medium.

### Detection of cDNA by microchip electrophoresis

In order to validate the results from microarrays, six ORFs (XF0358, XF0671, XF0785, XF1426, XF1937 and XF2688) were analyzed for RT-PCR by using microchip electrophoresis. The cDNAs from two different conditions (XDM_2_ and BCYE media) were synthesized and used in PCR with specific primers (Table 1). The Agilent 2100 Bioanalyzer separated the RT-PCR amplicons and quantified the expression of each gene. As expected, the ratios observed in this experiment were similar to those obtained through the microarray technique. Under XDM_2_ conditions, the ORFs XF0358, XF0785 and XF2688 showed a higher expression in this medium. Similar results were shown in the BCYE medium for ORFs XF1937, XF1426 and XF0671 (Figure 1). It is important to stress that the coefficient of variance between the replicates for each gene under the conditions studied was seen to be between 2.68 and 5.19%, demonstrating the high level of reliability of the results (Table 1).

## Figures and Tables

**Table 1:** Nucleotide sequences of the primer used to detect cDNA, fluorescence and the coefficient of variation obtained in microchip electrophoresis.

Gene ID^a^	Primers	Fragment (bp)	Fluorescence (RFU)^b^				CV (%)^e^		
			BCYE	XDM_2_	Log ratio^c^		BCYE	XDM_2_
XF0358	F- 5' AGCAACGGAGATAATTCG 3' R- 5' TTCACGCCTACCTTTTTC 3'	525	3.38	99.31	-1.46		4.17	2.74
XF0671	F- 5' GGTGAGGTTGCGTTAGTG 3' R- 5'ATCATTGCGTACACCCTC 3'	588	154.53	-	-^d^		3.13	-
XF0785	F- 5' GCATTGAAAACGGGTAAC 3' R- 5' TCAGATTGTTTGACGCTG 3'	886	-	96.21	-		-	5.19
XF1426	F- 5' GCGTCGGCTGCGCCATAG 3' R- 5' GATGTTAGCGATCTTGGG 3'	358	84.95	13.03	+0.81		3.50	2.68
XF1937	F- 5' GAACATAAAGCAGGCCAC 3' R- 5' GAGAGGCTCGAATTGATG 3'	702	141.38	-	-		2.99	-
XF2688	F- 5' GTAACACGGCAGGAAAAC 3' R- 5' AAGCCATGGCAGTAGAAG 3'	441	32.97	196.43	-0.77		4.59	3.56

^a^Simpson *et al.* (2000); ^b^Rate of fluorescence (RFU) obtained from replicate media; ^c^Log expression ratios of measured transcript levels determined for the two cultures. The log expression ratio is positive for genes that were more highly expressed in BCYE medium. The log is negative for genes that were more highly expressed in XDM_2_ medium; ^d^Genes that expressed only one condition and ^e^Coefficient of Variation (CV).

**Table 2:** Components in BCYE and XDM_2_ media, developed for *X. fastidiosa*.

Components	BCYE	XDM_2_
Glucose (10 g/L)	-^a^	+^b^
K_2_HPO_4_ (2.1 g/L)	-	+
KH_2_PO_4_ (0.8 g/L)	-	+
MgSO_4_ 7H_2_O (0.4 g/L)	-	+
Ferric pyrophosphate (0.125 g/L)^c^	+	+
Aces buffer (10 g/L)	+	-
Activated charcoal (2 g/L)	+	-
Yeast extract	+	-
L-cysteine (0.4 g/L)	+	-
L-serine (0.4 mg/mL)	-	+
L-asparagine (1.0 mg/mL)	-	+
L-methionine (0.4 mg/mL)	-	+
L-glutamine (4.0 mg/mL)	-	+
Vitamin stock solution (10 mL/L)^d^	-	+
Biotin (0.2 mL/L)	-	+
Phenol red (0.1%)	-	+

^a^(-) components taken from the media, ^b^(+) components added to the media, ^c^in the BCYE media, the ferric pyrophosphate concentration was 0.25 g/L and the ^d^Vitamin stock solution (10 mL/L) contained 0.2 mg _D_-biotin, 10 mg thiamine, 10 mg pyridoxine hydrochloride, 5.0 mg nicotinic acid, 0.05 mg vitamin B12 and 350 mg myo-inositol.

**Table 3:** *Xylella fastidiosa* genes induced in BCYE and XDM_2_ media (q-value^a^ (%) = 0.27 for all genes).

Gene ID	Gene name	Description	Log ratio^b^
Energy metabolism			
XF0869	*pdhB*	Dihydrolipoamide acetyltransferase	0.98^c^
XF0824	*pykA*	Piruvate kinase typo II	0.72
XF1855	*fumB*	Fumarate hydratase	-2.02^d^
XF0274	*pfkA*	6-phosphofrutokinase	-1.78
XF0457	*gapA*	Glyceraldehyde-3-phosphate dehydrogenase	-1.74
XF0292	*acnB*	Aconitate hydratase 2	-1.35
XF0909	*petB*	Ubiquinol cytochrome C	-1.30
XF1144	*atpG*	ATP synthase	-1.29
XF0557	*az*1	Electron transfer protein azurin I	-1.28
XF0303	*tpiA*	Triosephosphate isomerase	-1.27
XF1746	*yahK*	Alcohol dehydrogenase	-1.24
XF0183	*gcvT*	Glycine cleavage T protein	-1.24
XF1211	*mdh*	Malate dehydrogenase	-0.97
XF0258	*rfbC*	DTDP-4-keto-L-rhamnose reductase	-0.87
XF1550	*odhA*	Oxoglutarate dehydrogenase	-0.72

Transport			
XF1527	*xpsD*	General secretory pathway protein D precursor	1.04
XF0806	*secA*	Preprotein translocase SecA	0.98
XF1937	*gltP*	Proton glutamate symport	0.94
XF1913	*tatD*	Type V secretory pathway	0.80
XF1426	dr0830	Ion transporter	0.69
XF1728	f451	Transport protein	-3.27
XF0785	*sac*1	Sulfer deprivation response regulator	-2.41
XF1067	*algS*	Sugar ABC transporter ATP-binding protein	-1.42
XF0320	*citN*	Mg^2+^/ citrate complex transporter	-1.39
XF2455	*ccmA*	Heme ABC transporter ATP-binding protein	-1.32
XF2446	*malG*	ABC transporter sugar permease	-1.30
XF0874	*yecS*	ABC transporter permease	-1.14
XF0933	*feoB*	Ferrous iron transporter protein B	-1.13
XF0324	*afuA*	Periplasmic iron-binding protein	-0.99
XF2685	*sppA*	Protease IV	-0.94
XF1172	*secY*	Preprotein translocase SecY	-0.93
XF1476	*ynhE*	ABC tranporter membrane	-0.84
XF1520	*xpsH*	General secretory pathway protein H precursor	-0.81
XF2133	*yheS*	ABC transporter ATP-binding protein	-0.77

Membrane components and surface structure			
XF0343	*mopB*	Outer membrane protein	1.56
XF0373	*pilQ*	Fimbrial assembly protein	0.87
XF0103	dc14	Membrane protein	-1.22
XF1118	*murD*	UDP-N-acetylmuramoylalanine-D- glutamate ligase	-1.08
XF0881	*-*	D-alanil-D-alanina carboxipeptidase	-1.04
XF0478	*pilY*1	Fimbrial assembly protein	-1.11
XF0372	*pilP*	Fimbrial assembly protein	-1.04
XF1309	*mreB*	Rod shape-determining protein	-0.73
XF2542	*-*	Fimbrial protein	-1.01
XF0078	*mrkD*	Fimbrial adhesin precursor	-0.92
XF1632	*pilU*	Twitching motility protein	-0.69

RNA, DNA and nucleotide metabolism			
XF0935	*ilaIIA*	Methyltransferase	0.80
XF0822	*gltX*	Glutamyl-tRNA synthetase	0.78
XF0587	*purM*	5'-phosphoribosyl-5-aminoimidazole synthetase	0.62
XF0169	*tyrS*	Tyrosyl-tRNA synthetase	0.59
XF2178	*holA*	DNA polymerase III, delta subunit	-1.85
XF0223	*tgt/vacC*	Queuine tRNA-ribosyltransferase	-1.70
XF1909	*mutY*	A/G-specific adenine glycosylase	-1.23
XF2672	*purE*	Phosphoribosylaminoimidazole carboxylase, catalytic subunit	-1.10
XF0354	*recG*	ATP-dependent DNA helicase	-0.89
XF0676	*holB*	DNA polymerase III, delta subunit	-0.66
XF0549	*metG*	Methionyl-tRNA synthetase	-0.62
XF1297	SCF 11.04	Gluconolactonase precursor	-0.60

Biosynthesis of amino acids and proteins			
XF2465	*metA*	Homoserine O-acetyltransferase	0.71
XF1427	*argM*	Succinylornithine aminotransferase	0.69
XF1944	*dcp*	Peptidyl-dipeptidase	0.64
XF1189	*lon*	ATP-dependent serine proteinase La	0.62
XF1116	*lysA*	Bifunctional diaminopimelate	-2.17
XF0267	*pspB*	Serine protease	-1.02
XF2219	*hisD*	Histidinol dehydrogenase	-0.81
XF0624	*aroE*	Shikimate 5-dehydrogenase	-0.75
XF2324	*aroE*	3-phosphoshikimate 1-carboxyvinyltransferase	-0.71
XF1915	*trpG*	Anthranilate synthase component II	-0.64

Biosynthesis of cofactors, prosthetic groups and regulatory functions			
XF0956	*thiL*	Thiamine-monophosphate kinase	0.93
XF2592	*phoR*	Two-component system, sensor protein	0.83
XF0017	*hemF*	Coproporphyrinogen III oxidase, aerobic	0.82
XF0064	*bioB*	Biotin synthase	-1.52
XF0230	*panC*	Pantoate-beta-alanine ligase	-1.30
XF0322	*tctD*	Two-component system, regulatory protein	-1.20
XF0189	*bioA*	Adenosylmethionine-8-amino-7-oxononanoate aminotransferase	-1.05
XF0912	*sspB*	Stringent starvation protein B	-0.90
XF1626	*algR*	Two-component system, regulatory protein	-0.86
XF0911	*sspA*	Stringent starvation protein A	-0.71
XF2336	*colR*	Two-component system, regulatory protein	-0.70
XF0950	*ribD*	Riboflavin-specific deaminase	-0.69
XF0228	*folK*	2-amino-4-hydroxy-6-hydroxymethyldihydropteridine pyrophosphokinase	-0.66
XF2306	*hemB*	Delta-aminolevulinic acid dehydratase	-0.64
XF2545	*pilR*	Two-component system, regulatory protein	-0.63
XF0953	*ribA*	GTP cyclohydrolase II/3,4-dihydroxyl-2-butanone 4-phosphate synthase	-0.60

Biosynthesis of fatty acids and phospholipids			
XF0671	*fabG*	3-oxoacil-[ACP] reductase	0.71

Degradation of molecules			
XF1965	*dhaA*	Haloalkane dehalogenase	0.84
XF1743	*est*	Esterase	-1.16
XF1253	*lipP*	Lipase	-0.64

Toxins			
XF1029	*gaa*	Glutaryl-7-ACA acylase precursor	-2.69
XF2759	*frpC*	Haemolysin-type calcium binding protein	-0.73
XF1220	*cvaB*	Colicin V secretion ABC transporter ATP-binding protein	-0.70

Related to plasmid			
XFa0047	*taxC*	Nickase	-0.70

Related to phage			
XF2478	*int*	Phage-related integrase	-0.75

Cell division			
XF0796	*ftsW*	Cell division protein	-0.81

Others			
XF0961	*bcp*	Bacterioferritin comigratory protein	-1.32

Hypothetical and conserved proteins			
XF0473	-	Hypothetical protein	1.16
XF0497	rv2514c	Conserved hypothetical protein	0.74
XF1620	-	Hypothetical protein	0.69
XF0374	-	Hypothetical protein	0.67
XF2041	-	Hypothetical protein	0.67
XF1252	b2520	Conserved hypothetical protein	0.66
XF0597	dr1792	Conserved hypothetical protein	0.64
XF2734	-	Hypothetical protein	0.59
XF1812	dr0620	Hypothetical protein	-2.95
XF1240	-	Hypothetical protein	-2.50
XF0172	-	Conserved hypothetical protein	-2.46
XF1753	-	Hypothetical protein	-2.32
XF2688	-	Conserved hypothetical protein	-2.28
XF0358	-	Hypothetical protein	-2.07
XF2687	-	Hypothetical protein	-1.70
XFa0028	-	Hypothetical protein	-1.57
XF0272	-	Conserved hypothetical protein	-1.39
XF0201	-	Conserved hypothetical protein	-1.35
XF2428	-	Conserved hypothetical protein	-1.19
XF1117	-	Conserved hypothetical protein	-1.16
XF1086	-	Conserved hypothetical protein	-1.15
XF1798	-	Hypothetical protein	-1.14
XF2510	-	Hypothetical protein	-1.13
XF0675	hi0457	Conserved hypothetical protein	-1.09
XF2400	-	Conserved hypothetical protein	-1.05
XF0601	-	Conserved hypothetical protein	-1.05
XF2449	-	Conserved hypothetical protein	-1.02
XF2008	tm1181	Conserved hypothetical protein	-1.01
XF0638	-	Hypothetical protein	-1.01
XF2074	-	Conserved hypothetical protein	-0.68
XFa0018	-	Hypothetical protein	-0.93
XF2023	-	Conserved hypothetical protein	-0.67
XF1881	-	Hypothetical protein	-0.67
XF0357	-	Hypothetical protein	-0.64
XF1323	-	Hypothetical protein	-0.62
XF2647	-	Conserved hypothetical protein	-0.61
XF2363	-	Conserved hypothetical protein	-0.61
XF2427	-	Conserved hypothetical protein	-0.60
XF1854	*ctp*	Hypothetical protein	-0.59
XF0766	-	Hypothetical protein	-0.52

^a^q-value is the lowest False Discovery Rate at which the gene is called significant and measures how significant the gene is: as *d*_*i*_ > 0 increases, the corresponding q-value decreases (Tusher *et al.*, 2001), ^b^Log expression ratios of measured transcript levels determined for the two cultures; ^c^The log expression ratio is positive for genes that were more highly expressed in BCYE medium and ^d^The log is negative for genes that were more highly expressed in XDM_2_ medium.
